# IMPLEMENTATION OF VIRTUAL REALITY TO IMPROVE QUALITY OF LIFE FOR VETERANS IN HOSPICE AND PALLIATIVE CARE

**DOI:** 10.1093/geroni/igad104.3420

**Published:** 2023-12-21

**Authors:** Alexandra Laffer, Melanie Corle, Anastasia Canell, Lauren Moo, Megan Gately, Kristen Dillon

**Affiliations:** Edith Nourse Rogers Memorial VA Medical Center (VA Bedford), Bedford, Massachusetts, United States; Edith Nourse Rogers Memorial VA Medical Center (VA Bedford), Bedford, Massachusetts, United States; VA Bedford Healthcare System, Bedford, Massachusetts, United States; VA Bedford Healthcare System, Bedford, Massachusetts, United States; VA Bedford Healthcare System, Bedford, Massachusetts, United States; Edith Nourse Rogers Memorial VA Medical Center (VA Bedford), Bedford, Massachusetts, United States

## Abstract

Virtual Reality (VR) is a safe and effective adjunctive care option with multiple clinical applications. An interdisciplinary VR program aimed at improving quality of life for veterans receiving inpatient hospice and palliative care was implemented at the VA Bedford Health Care System. Principles from the 4Ms of Geriatrics (Mentation, Mobility, Medications, and Matters Most) guided evaluation and outcomes. Implementation involved decision-making around hardware and software, learning about processes at other facilities, financing, and creating policies and procedures. Project development began in 2021 and data collection began in March 2023. Veteran residents enrolled in inpatient hospice and palliative care were invited to participate. As of August 2023, 22 VR visits were conducted with 13 veterans. Preliminary data suggests VR can be used with a wide range of individuals receiving hospice and palliative care. Analysis of pre-post measures indicate improvement in mood (62%) and overall day (81%), and reduced pain. Barriers to engagement include staffing (e.g., availability), technology (e.g., VR technical issues), hospital procedures/policies (e.g., infection control, COVID-19), and patient factors (e.g., interest, symptoms). Facilitators include hospital support, interdisciplinary collaboration, and understanding of the setting and patient preferences/ needs. Although conception to implementation took two years, a VR program to improve quality of life for veterans enrolled in hospice and palliative care appears to be feasible and clinically beneficial. Experience has shown that flexibility, adaptability, and an individualized approach facilitate engagement. Best practices in the application of VR within the hospice and palliative care setting will continue to be evaluated and honed.

